# SISTEM: simulation of tumor evolution, metastasis, and DNA-seq data under genotype-driven selection

**DOI:** 10.1093/bioinformatics/btaf634

**Published:** 2025-11-23

**Authors:** Samson Weiner, Mukul S Bansal

**Affiliations:** School of Computing, University of Connecticut, Storrs, CT 06269, United States; School of Computing, University of Connecticut, Storrs, CT 06269, United States; Institute for Systems Genomics, University of Connecticut, Storrs, CT 06269, United States

## Abstract

**Summary:**

SISTEM is a software package and mathematical framework for simulating tumor evolution and cell migrations at single-cell resolution. Unlike existing frameworks which simulate cancer cell populations under the neutral coalescent or using simple birth–death models, SISTEM simulates tumor populations under somatic clonal selection using an agent-based framework. SISTEM can generate mutation profiles, read counts, and DNA sequencing reads along with ground truth cell lineages and migration graphs under a number of easily customizable mutation and selection models. For improved realism, SISTEM allows for cell fitness to be driven by genomic events of various scales including single nucleotide variants, segmental gains and losses, whole-chromosomal and chromosome-arm aberrations, and whole-genome duplications. SISTEM also includes numerous migration models to simulate metastatic cancers, facilitating the exploration and evaluation of diverse migration patterns.

**Availability and implementation:**

SISTEM is written in Python and is freely available open-source under GNU GPLv3 from: https://github.com/samsonweiner/sistem

## 1 Introduction

Genetic heterogeneity within and across tumor cell populations is a pervasive feature of human cancers (Burrell *et al.* 2013). Clonal evolution models explain observed genetic diversity to be the result of a continuous accumulation of somatic mutations occurring over many cell replication cycles (McGranahan and Swanton 2015). Increasingly available genetic data, made possible by advances in sequencing technologies, have made apparent the complexity of clonal evolution and the influence of selection induced by the tumor microenvironment in shaping mutational and phenotypic patterns (Lawson *et al.* 2018, Coorens *et al.* 2021). The same is true for metastasis, itself an evolutionary process shaped by strong selective pressures (Obenauf and Massagué 2015), with recent analyses suggesting complex cell migration patterns (Turajlic and Swanton 2016).

Our ability to study cancer evolution benefits from the continued development of improved computational methods to profile somatic mutations from DNA sequencing data and to reconstruct evolutionary history (Beerenwinkel *et al.* 2015). Given the wide range of available tools, and the lack of known ground truth in real datasets, simulations are critical for measuring and benchmarking their performance. This has led to the development of numerous dedicated simulation frameworks for modeling tumor evolution and next-generation sequencing (NGS) data (Qin *et al.* 2015, Chu *et al.* 2017, Yang *et al.* 2019, Posada 2020, Feng and Chen 2022, Mallory and Nakhleh 2022, Srivatsa *et al.* 2023, Weiner and Bansal 2023). However, the majority of existing simulation frameworks simulate the cell lineage under the coalescent, whereby the topology is generated prior to adding mutations, while the others utilize a simple branching process which simulates the topology and mutations simultaneously. In either case, the approach used by existing frameworks is limited in its ability to model selection despite its crucial role in promoting tumor progression and metastasis.

One way to address the above limitation is to use an agent-based evolutionary framework to model selective forces in growing cell populations. Agent-based models simulate simple behaviors and interactions of a population of autonomous agents in order to study the complex properties of the population, and have already been widely used to model processes related to cancer evolution, tumor microenvironments, immune response, and other biological phenomena. This includes various processes consequential to tumor morphology including growth dynamics (Reiter *et al.* 2013, Chen *et al.* 2015, Harris *et al.* 2019), cell motility and invasion (Basanta *et al.* 2008, Kolev and Zubik-Kowal 2011, Letort *et al.* 2019), and mutation rates and selective forces (Bozic *et al.* 2010, Valind *et al.* 2013, Salehi *et al.* 2021, Dinh *et al.* 2025). See Metzcar *et al.* (2019) for a review of agent-based modeling in cancer. Despite providing a more realistic framework for simulating tumor cell lineages, agent-based models have not yet been used in any standalone DNA-seq simulator. This represents a significant research gap since selection plays an important role in cancer progression.

In this work, we present SISTEM (SImulation of Single-cell Tumor Evolution and Metastasis), a new mathematical framework and software package for simulating tumor growth, metastasis, and DNA-seq data under genotype-driven selection. SISTEM models tumor clones as individual agents which proliferate, mutate, and migrate over discrete generations, competing with other clones occupying the same tissue. By using an agent-based model, SISTEM bridges the gap between DNA-seq simulators and simulators used to study specific complex behaviors in tumors. SISTEM also implements numerous enhancements that improve biological realism and utility, combining many useful features that appear in separate frameworks under a single unified tool. Some key features and capabilities of SISTEM include:

1. The use of a dynamic agent-based model to simulate tumor growth under tissue-specific selection landscapes, coupled with several algorithmic enhancements to improve efficiency.
2. A novel migration model capable of simulating various migration patterns in metastatic cancers, incorporating explicit distances and priors reflecting organotropism.
3. A diverse mutation model which considers both single nucleotide variants (SNVs) and four distinct copy number aberration (CNA) mechanisms.
4. The ability to generate synthetic data from numerous points along the DNA-seq analysis pipeline, including mutation profiles, read count matrices, and raw allele-specific DNA-sequencing reads.

## 2 Features and implementation

**Figure 1.**
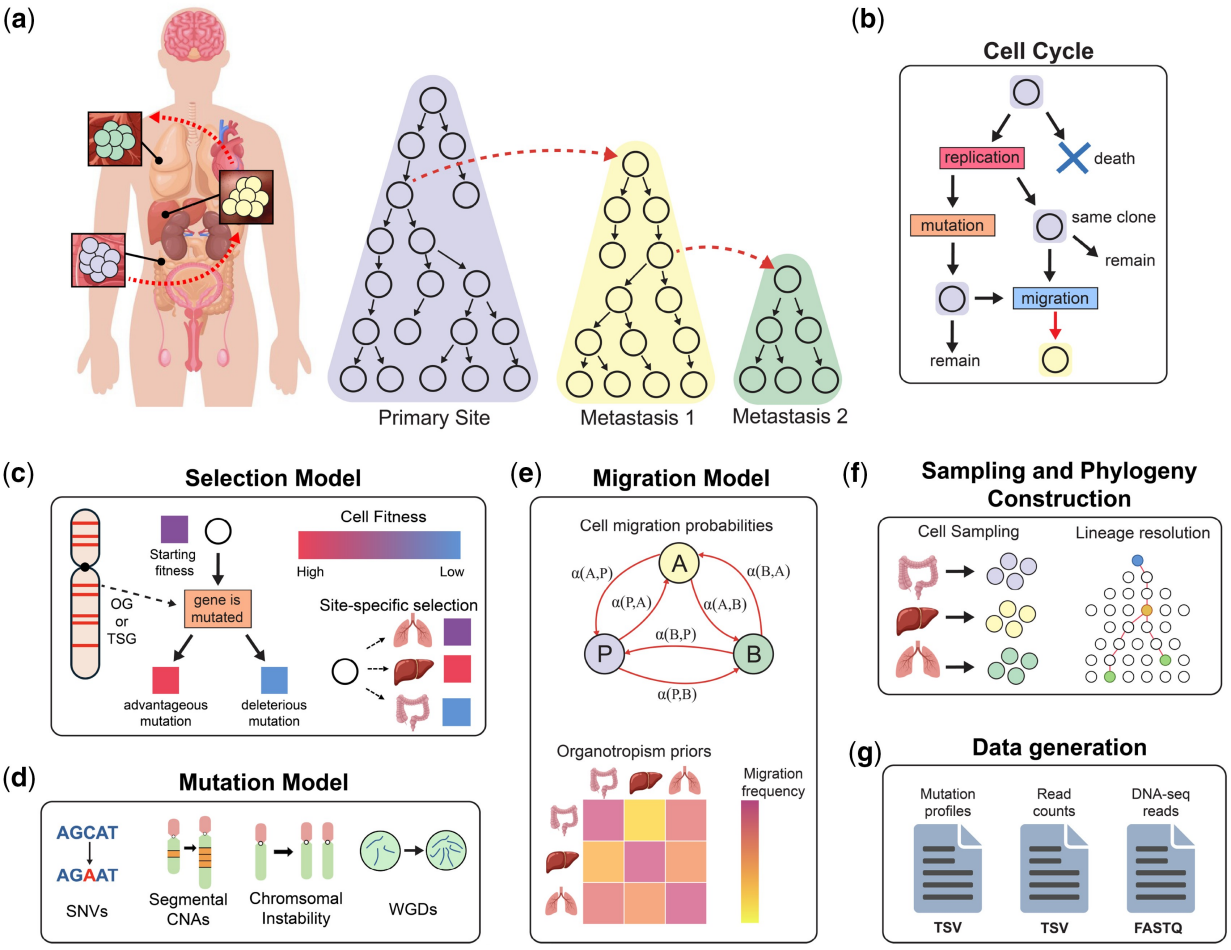
Overview of the SISTEM model. (a) SISTEM simulates the evolution of cancer cell populations as separate lineages in distinct anatomical locations that are linked by cell migrations. (b) Tumor growth and cell migrations occur over discrete generations through a birth–death-migration process. At the end of an exponentially distributed lifespan, every cell either replicates or dies, with the daughter replicating cells potentially acquiring new mutations. Additionally, at each generation every surviving cell has a chance to migrate to a new anatomical site. (c) The probability that a cell replicates depends on the cell’s fitness, which is computed according to the selection model. In SISTEM, the selection model determines how mutations increase or decrease fitness based on gene- or chromosome arm-level selection coefficients. These coefficients can vary from site-to-site, leading to diverse site-specific fitness landscapes. (d) A variety of mutations are possible in SISTEM including SNVs and four distinct CNA mechanisms: segmental amplifications and deletions, chromosome-arm missegregation, whole-chromosomal missegregation, and whole-genome duplications (WGDs). (e) The probability that a cell migrates to a different anatomical site is computed according to the migration model. These probabilities can reflect underlying organotropism priors and/or the compatibility of the cell’s genotype with the fitness landscape of the destination. (f) After each tumor reaches a critical population size, SISTEM samples cells from each site and constructs high-resolution lineages at the clonal or single-cell level. Passenger mutations are added at this stage. (g) Various types of output data can be generated for the observed cells or clones, including ground truth phylogenies and migration graphs, CNA and/or SNV profiles, and bulk or single-cell read counts and synthetic DNA-seq reads.

A graphical overview of the SISTEM simulation framework is shown in Fig. 1. SISTEM models the somatic evolution of individual cancer cells located in one or multiple anatomically distinct tumors and the migrations between tumors over discrete generations as a stochastic branching process (Durrett and Durrett 2015). Cells are characterized by two properties: an anatomical site *a*, describing the cell’s location, and a genome *G*, consisting of the cell’s allele-specific copy number profiles (CNPs) and a set of SNVs. SISTEM utilizes the simplified genome representation from CNAsim (Weiner and Bansal 2023), optimizing for scalability while preserving gene location. See Section S.1, available as supplementary data at *Bioinformatics* online for details on cell properties. At the end of a cell’s lifespan, the cell either dies off or replicates into two potentially mutated daughter cells. The lifespan of a cell can either be fixed at a single generation, or can be drawn from an exponentially distribution and parameterized by an input turnover rate (Pompei and Cosentino Lagomarsino 2023). Following the cell replication/death events, each surviving cell has the potential to colonize or migrate to other anatomical sites.

*Selection and mutation.* The probability that a cell replicates (and migrates, depending on the model; see below) is determined by the cell’s fitness, computed as a function of its genome and site, and scaled according to a predetermined population growth dynamic. See Section S.2, available as supplementary data at *Bioinformatics* online for details on cell replication. Selection is modeled using a multiplicative fitness landscape that is unique to each anatomical site. SISTEM offers three baseline selection models, but allows users to create custom models for flexible fitness landscapes. Two CNP-based selection models are adapted from (Dinh *et al.* 2025) and compute fitness from copy numbers only. The first CNP model, named the “region” model, assigns a selection coefficient δ to each region, where δ>1 corresponds to oncogenes (OGs), δ<1 corresponds to tumor suppressor genes (TSGs), and δ=0 are neutral. Amplifications to OGs (resp. TSGs) increase (resp. decrease) cell fitness, while deletions have the opposite effect. The second CNP model, named the “chromosome-arm” model, works similarly but assigns a selection coefficient to each chromosome-arm instead of regions, where the coefficients reflect the relative balance of OGs to TSGs on that arm (Davoli *et al.* 2013). The last selection model, named the “hybrid” model, extends the region model to consider SNVs. In particular, consequential SNVs occurring in a TSG or OG add an additional multiplier to the relative fitness effect of that region, while SNVs occurring in neutral regions with essential genes can be deleterious, decreasing fitness (Woo and Li 2012). For additional details on the selection models, see Section S.3, available as supplementary data at *Bioinformatics* online. To facilitate change in the fitness landscape over time, SISTEM implements SNVs and four distinct CNA mechanisms: segmental amplifications and deletions, chromosome-arm missegregation, whole-chromosomal missegregation, and whole-genome duplications (WGDs). Consistent with the underlying biological mechanisms (Knouse *et al.* 2017, Harbers *et al.* 2021), the latter three CNA events result in new chromosomal strands. Section S.4, available as supplementary data at *Bioinformatics* online contains further details on the mutation model. To ensure stability in the fitness landscape and cell properties, each cell is subject to viability checkpoints upon acquiring new mutations and will immediately die if any conditions are failed; see Section S.5, available as supplementary data at *Bioinformatics* online.

*Cell migration.* A key novel feature of SISTEM is the inclusion of an explicit migration model to simulate metastatic cancers. The probability that a cell migrates from its current site *a* to a different site *b* is the product of a baseline per-generation migration probability and the inverse of an abstract distance function d(a,b), defined according to the migration model. SISTEM provides two migration models, but allows users to define custom models. In the “static” model, *d* is defined according to precomputed (or randomly generated) pairwise distances which are constant across all cells and generations. This can be interpreted as an organotropism term (Gao *et al.* 2019), e.g. compatibility with organ microenvironments (Chen *et al.* 2018), transportation pathways (Pretzsch *et al.* 2019), or vascular and nutrient supply (Obenauf and Massagué 2015). In the “genotype” model, *d* is defined separately for each cell and decreases as the cell acquires mutations which are advantageous in the target site (Sha *et al.* 2020). Thus, the migration rate of a cell increases as the cell becomes more compatible with the target site’s fitness landscape. Section S.6, available as supplementary data at *Bioinformatics* online provides additional details.

*Cell sampling and cell lineage generation.* As tumor cell population size is in the order of $10^{6}-10^{9}$ during malignant growth (Beerenwinkel *et al.* 2007), creating an agent for each individual cell becomes computationally infeasible. SISTEM dramatically increases efficiency without sacrificing complexity by simulating agents at the clonal level, where a clone is defined as the set of cells with an identical sequence of driver mutations. This follows from the fact that cells from the same clone share replication, mutation, and migration probabilities. See Section S.7, available as supplementary data at *Bioinformatics* online for details on the simulation algorithm. The cost of this optimization is the inability to simulate passenger mutations and cell-to-cell variability during the branching process. To reintroduce passenger mutations and resolve single-cell lineages, SISTEM performs a second simulation stage after threshold population sizes are reached in each anatomical site using the “down-up-down” technique (Dinh *et al.* 2025). First, cells are sampled from the wider population of each anatomical site and a clone tree is constructed from the observed clones by tracing backwards. Second, bifurcations and multifurcations in the clone tree are fully resolved as a coalescent where ancestral population sizes follow clonal cell counts recorded in the first stage. Once resolved, passenger mutations are added in a top-down traversal of the cell lineage tree. For additional details on cell sampling and cell lineage tree construction, see Section S.8, available as supplementary data at *Bioinformatics* online.

*Performance and scalability.* SISTEM is highly scalable and can simulate thousands of distinct clones totaling millions of cells across multiple anatomical sites within minutes on commodity hardware. Table 1 reports the running time and memory usage of SISTEM for different numbers of cells and anatomical sites.

**Table 1.** Resource requirements of SISTEM in terms of total runtime in minutes and memory in MB.^a^

Min detectable		1 Site	3 Sites	5 Sites
50 000	Runtime	0.04 min	6.67 min	18.02 min
	Memory	99 MB	895 MB	1098 MB
500 000	Runtime	0.46 min	20.31 min	25.27 min
	Memory	177 MB	1031 MB	1157 MB

a The column labeled min detectable corresponds to the minimum number of cells that must be present in each site for the simulation to terminate. These experiments used SISTEM’s chromosome-arm selection model with parameters nsite∈{1,3,5}, and default values of other parameters. We used the fixed-distance anatomy model with site-specific selection libraries, with the two farthest sites differing in their selection coefficients by approximately 20%. All results are averaged over 25 runs, and all experiments were executed on a laptop computer with an Apple M3 processor and 16 GB of RAM.

## 3 Availability, requirements, and usage

SISTEM is implemented in Python and can be used on all platforms with a Python interpreter. It can be easily installed using anaconda or pip. By default, SISTEM requires no input files, only appropriate selection of models and parameters. Generating synthetic sequencing reads requires the user to pass one or multiple reference genomes and the additional installation of a few commonly used bioinformatics tools. SISTEM is open source under GNU GPLv3. Source code, documentation, and example experiments are freely available from https://github.com/samsonweiner/sistem.

*Types of data generated.* The data generated by SISTEM includes a detailed account of the population size, clonal composition, and fitness landscape of each tumor, which can be paused and explored at any given generation. Outside of a ground truth cell lineage tree, clone tree, and migration graph, SISTEM is also capable of generating DNA-seq data and its derivatives of the observed cells in the sample. This includes cell or clone-specific CNPs and SNV profiles, read counts, and raw single-cell DNA-seq reads. In the latter case, SISTEM constructs a full reference sequence for each mutated cell and computes a coverage distribution before calling a third party short read simulator. See Section S.9, available as supplementary data at *Bioinformatics* online for further details on data generation.

*Example experiments.* To demonstrate the features of SISTEM, we showcase two experiments: One involving the evaluation of a computational method for inferring tumor cell evolutionary histories, and another experiment that demonstrates how simulated datasets can be parameterized to mimic cancer subtype-specific mutation patterns. In the first experiment, we assess the performance of DICE-bar (Weiner and Bansal 2025), a recently published method for reconstructing tumor cell lineage trees using CNA profiles at single-cell resolution. Details on this experiment appear in Section S.10, available as supplementary data at *Bioinformatics* online. In the second experiment, we first analyze real genomic data from the MSK-MET pan-cancer cohort (Nguyen *et al.* 2022) to obtain cancer subtype-specific mutation patterns, then generate tailored simulated datasets for each subtype. Details on this experiment appear in Section S.11, available as supplementary data at *Bioinformatics* online.

## 4 Discussion and conclusion

SISTEM is a software package for improved simulation of tumor growth, metastasis, and DNA-seq data. Unlike previous simulators, SISTEM uses an agent-based model to capture the effects of selection in shaping population landscapes and migrations, improving biological realism, and offers a novel migration model to simulate metastatic cancers. As demonstrated through the two example experiments, SISTEM can facilitate the rigorous benchmarking of methods used to study various aspects of tumor evolution, and its parameters can be customized to create simulated datasets representative of specific cancer subtypes. Another possible application of SISTEM is parameter estimation. Some recent studies have used simulations to better understand the selective forces and mutation rates in real tumors, highlighting the promise of simulation-based parameter estimation for predicting tumor progression and stratifying patient subtypes (Xiang *et al.* 2024, Dinh *et al.* 2025). However, while manual parameterization of isolated parameters can lead to reasonable approximations of real data for the purposes of benchmarking and method evaluation, the complex parameter space of the SISTEM model suggests that a more sophisticated parameter inference framework is likely necessary to facilitate highly accurate and robust simulation-based parameter estimation. One potential parameter inference framework which shows promise is Neural Posterior Estimation (NPE) (Dyer *et al.* 2024), a neural network-based Bayesian inference model that has found success in other problems with high-dimensional parameter spaces.

While the SISTEM simulation model is designed for improved biological realism over existing simulators, it has several important limitations that could be addressed in future work. These limitations can be broadly grouped into four categories. First, SISTEM makes several simplifying assumptions related to population growth and mutations which, while reducing the parameter selection burden on the user, neglect complex phenomena. These assumptions include a strict logistic population growth curve without consideration of treatment responses or similar effects, and a simplified selection landscape that does not consider the interactions or cascading effect of mutations. Additionally, SISTEM uses constant mutation rates across lineages, and does not model some small or rare mutation events such as structural variants, indels, and breakage-fusion bridge cycles. Second, SISTEM does not capture the complex interactions at play in the tumor microenvironment. Access to resources, nutrient gradients, immune cell response, interaction with healthy tissue, and hypoxia are among many factors which influence selective pressures and thus growth patterns and mutation signatures. SISTEM also ignores any notion of intra-tumor cellular location, which can be used to model cell motility and other effects through the use of a 2D or 3D lattice (Harris *et al.* 2019). Third, while mutation profiles and single-cell DNA-sequencing data can be generated with SISTEM, including other data modalities, e.g. RNA-sequencing, would widen the breadth of possible applications. And fourth, SISTEM can be computationally demanding, which can limit its applicability in some cases. For example, while simulating agents at the clonal level leads to tremendous speedup, runtime can still be a bottleneck for experiments involving many anatomical sites. Careful parallelization of SISTEM simulations could help address this limitation.

## Supplementary Material

btaf634_Supplementary_Data

## Data Availability

SISTEM software is available open-source from https://github.com/samsonweiner/sistem. An archival copy of the SISTEM software version used in this work is available from Zenodo with the following DOI: 10.5281/zenodo.17656279
