# Supplementary material for: SISTEM: simulation of tumor evolution, metastasis, and DNA-seq data under genotype-driven selection
Source: Bioinformatics. 2025 Nov 23;41(12):btaf634. doi: 10.1093/bioinformatics/btaf634 (PMC12701798; doi:10.1093/bioinformatics/btaf634)
Supplement: btaf634_Supplementary_Data [file btaf634_supplementary_data.pdf]

Supplementary Text

S.1 Cell genome representation and properties

Each cell is characterized by two properties: an anatomical site a and genome G. The anatomical site a is the integer id of the site currently inhabited by the cell. For the genome, SISTEM utilizes a simplified representation consisting of a set of K chromosomes, where each chromosome k of reference length n_k is comprised of two data structures. First, the copy number profile (CNP) $X_k = \langle i \mid 0 \leq i \leq m_k \rangle$ of the chromosome is an array containing integer region ids corresponding to a unique M-length DNA segment from the reference sequence, where $m_k = \lceil n_k/M \rfloor$. For simplicity, we use $x_{k,i} \geq 0$ to denote the number of copies of the ith reference region on chromosome k. Here, we utilize the vector representation rather than tracking the copy numbers $x_{k,i}$ directly to preserve the location and order of regions. Second, the single-nucleotide variants (SNVs) of the chromosome are described by a set of integers $Y_k = \{j \mid 0 \leq j \leq n'_k\}$, where j corresponds to the presence of a non-reference nucleotide on the jth base pair and n'_k is the mutated (current) chromosome length. We use $y_{k,i}$ to denote the number of SNVs in region i of chromosome k.

S.2 Cell replication and death

Cells in each tumor population follow a birth-death process described in [4]. Cell lifespan is exponentially distributed with rate parameter γ, where upon the end of its lifespan, the cell either replicates or dies. At generation t, let the total number of cells in site a be denoted $N_a(t)$. Each cell divides with probability $p_c(t)$ or dies with probability $1 - p_c(t)$. For each cell c in site a whose lifespan ends at generation t, the probability that the cell replicates at time t is defined as

$$p_c(t) = \frac{s_a(c)}{\bar{s}_a} \cdot \Big(1 - \frac{N_a(t)}{E_a(t) + N_a(t)}\Big),$$

where $\bar{s}_a$ is the mean fitness rate of all $N_a(t)$ cells at time t. The term $E_a(t)$ is the expected number of cells in site a at time t and is used to conform the total cell count in a to follow some given population dynamic. The value of $E_a(t)$ is computed under a logistic growth curve:

$$E_a(t) = E_a(t-1) + g \cdot E_a(t-1) \cdot (1 - \frac{E_a(t-1)}{K_a}),$$

where g is the growth rate and K_a is a predetermined site-specific carrying capacity of the tumor. Because we expect cells to evolve considerably before migrating to new sites, we model the population dynamics of metastatic sites differently. In particular, given a max growth rate $g_{\text{max}} >= g$, upon the seeding of site a by cell c, we compute its growth rate as

$$g_a = g + \frac{\log(s_a(c) - \check{s}_a + 1)}{\log(\hat{s}_a - \check{s}_a + 1)} \cdot (g_{\text{max}} - g),$$

where $\check{s}_a$ and $\hat{s}_a$ correspond to the fitness of a non-mutated cell and maximally fit cell, respectfully.

S.3 Genotype-driven selection model

Selection in SISTEM is modeled using a multiplicative fitness landscape and allows for different selection parameters in different anatomical sites. The fitness $s_a(c) \in \mathbb{R}^+$ of cell c in site a is simply a function of its genome and site. The fitness of a cell can change as new mutations are acquired or if the cell migrates to a new anatomical site. SISTEM offers three baseline selection models, but the user is able to create custom models for flexible fitness landscapes. Two of the baseline models compute fitness using only CNPs, and are adapted from [4], while the last model computes fitness from both CNPs and SNVs. The following section defines each model in detail.

In the 'chromosome-arm' model, each arm of chromosome k is assigned a selection coefficient $\delta_{k,arm} \in (-1,1)$ reflecting the relative balance of oncogenes (OGs) to tumor suppressor genes (TSGs) along the arm. $\delta_{k,arm} > 0$ indicates the arm is weighted towards OGs, $\delta_{k,arm} < 0$ indicates the arm is weighted towards TSGs, and $\delta_{k,arm} = 0$ means the arm has an equal balance. Fitness is computed as

$$s_a(c) = \prod_{k=1}^{K} \prod_{arm \in \{p,q\}} (1 + \delta_{k,arm})^{x_{k,arm}/p_c},$$

where $x_{k,arm}$ is the average copy number of all reference regions located on that arm, i.e. $\frac{1}{m_k}\sum_{i=1}^{m_k} x_{k,i}$, and p_c is cell ploidy. This formulation follows from previous observations of frequent amplifications of arms with a high density of OGs and deletions of arms with a high density of TSGs [3].

The other CNP-based model, named the 'region' model, is similar to the first but operates on the gene level. In particular, each reference region i on chromosome k is assigned a selection coefficient $\delta_{k,i} \in (-1,1)$, where positive values correspond to OGs, negative values correspond to tumor suppressor genes TSGs, and zero corresponds to neutral regions (NEU). Fitness is then computed as

$$s_a(c) = \prod_{k=1}^{K} \prod_{i=1}^{m_k} (1 + \delta_{k,i})^{x_{k,i}/p_c}.$$

Note that amplifications of OGs and deletions of TSGs are advantageous, increasing the fitness, while deletions of OGs and amplifications of TSGs are deleterious, decreasing the fitness.

Note that the first two selection models consider only copy numbers when computing fitness. The third and last baseline model, named the 'hybrid' model, extends the region model to consider SNVs as well. The idea is that SNVs can disrupt the function of genes, thereby altering the selective effect of that gene on the cell's fitness. Here we distinguish between driver SNVs, which have this effect, and passenger SNVs, which have no effect (synonymous). For each region on each chromosome (k,i), we assign a second coefficient $\lambda_{k,i} \in (-1,1)$ where $\lambda_{k,i} > 0$ if (k,i) is a TSG but $\lambda_{k,i} < 0$ or $\lambda_{k,i} > 0$ is (k,i) is an OG. In addition to OGs and TSGs, SNVs can disrupt the function of neutral genes which are essential to the cell's basic functions and are therefore deleterious [11]. We designate a fraction of NEU regions to be essential genes (EG), where if (k,i) is an EG then $\delta_{k,i} = 0$ and $\lambda_{k,i} > 0$, while $\lambda_{k,i} = 0$ for the remaining neutral genes. Fitness is computed as

$$s_a(c) = \prod_{k=1}^{K} \prod_{i=1}^{m_k} \left(1 + \delta_{k,i} \cdot (e^{-\hat{y}_{k,i}\lambda_{k,i}})\right)^{x_{k,i}/p_c} \cdot \left(e^{-\hat{y}_{k,i}\lambda_{k,i}}\right)^{h(k,i)},$$

where $\hat{y}_{k,i}$ is the average number of driver SNVs on any copy of region i of chromosome k and h is an indicator function with $h(k,i) = 1$ if region (k,i) is an EG and $h(k,i) = 0$ otherwise. Note that the term $e^{-\hat{y}_{k,i}\lambda_{k,i}}$ is 1 when $\hat{y}_{k,i} = 0$ and approaches 0 as $\hat{y}_{k,i}$ increases. In other words, driver SNVs disrupting TSGs increase fitness, those disrupting EGs decrease fitness, and those disrupting OGs can have either effect. This follows from previous observations in which TSGs positively select for mutations which disrupt or inactive function, while OGs display both negative and positive selection for these mutations [1].

The selection coefficients (δ's and/or λ's) and gene classification labels can be provided directly by the user or generated randomly. In the latter case, coefficients are assigned as follows. In the chromosome-arm model, selection coefficients are randomly drawn from $(-\delta^{max}, \delta^{max})$, which is by default $\delta^{max} = 0.25$. In the region model, given oncogene rate d^{OG} and tumor suppressor rate d^{TSG}, we determine the behavior of each region (k,i) to be OG, TSG, or NEU with a multinomial

$$b_{k,i} \sim \text{Multinomial}(1; d^{og}, d^{tsg}, 1 - d^{og} - d^{tsg}).$$

If $b_{k,i} = \text{OG}$ then $\delta_{k,i}$ is drawn uniformly in the range $(0, \delta^{max})$, while if $b_{k,i} = \text{TSG}$ then the range is $(-\delta^{max}, 0)$. Lastly, $\delta_{k,i} = 0$ if $b_{k,i} = \text{NEU}$. The hybrid model extends this by selecting essential genes from

among those labeled NEU by simply drawing from a binomial with probability d^{ESS}. By default, all regions with negative λ are set to $-\lambda^{max}$, while all regions with positive λ are set to λ^{max}. By default, $\lambda^{max} = 0.1$. For TSGs and EGs, λ is always positive, while for OGs, the sign of λ is determined with a coin flip.

The selection model can assign unique selection coefficient libraries to anatomical site, creating a different fitness landscape in each. The user can provide precomputed selection coefficients for each site, or they can be generated randomly. In the latter case, this can be done one of two ways and in both cases is controlled by a variation parameter that tunes the mean number of drivers to alter site-to-site. We assume the selection library of the primary site a has been initialized normally and let D be the number of driver regions (regions with non-zero selection coefficients). The variation in fitness landscape is controlled by the user-defined parameter v defined as the mean ratio of drivers to alter. In the first approach, exactly D' drivers are altered for each metastatic site b, where

$$D' \sim \text{Binomial}(D; v).$$

For each site b independently, we select D' driver regions at random and alter their δ's as follows. For driver region (k, i), we redraw $\delta'_{k,i}$ uniformly in the range $(-\delta^{max}, \delta^{max})$.

This approach does not reflect any notion of organotropism, or similarities in tumor microenvironments. In certain instances, it may be desirable to have some fitness landscapes be more similar to others and vice-versa. To achieve this, we explore a second approach that alters the set of drivers to reflect a given pairwise distance matrix between sites. First, we draw D' as before and set this to be the number of drivers which differ between the two sites with the largest pairwise distance d_{max}. The number of drivers which differ between all pairs of sites is then determined by scaling D' by the ratio of the pairwise distance over d_{max}, rounded to integer values. Second, we assign an overlapping set of altered drivers to each site in accordance with the number of differing drivers between each pair determined previously.

S.4 Mutation mechanisms

After the birth-death process, the genomes of replicated cells may undergo new mutations. Suppose cell c replicates into daughter cells c_1 and c_2 at generation t. We set one daughter cell c_1 to be identical to its parent c, while the genome of c_2 mutated by one or multiple of the following events: focal (segmental) CNAs, SNVs, chromosome-arm CNAs, whole-chromosomal CNAs, and whole-genome duplications (WGDs). We denote the probability of each event occurring by p_{focal}, p_{SNV}, p_{arm}, p_{whole}, and p_{WGD}, respectively. SISTEM models a wide range of possible mutations by stochastically selecting event properties, for example the location, segment length, event type (amplification vs deletion), and amplification magnitude of focal CNAs are modeled with separate tunable parameters. We also distinguish between driver and passenger mutations, depending on the selection model; while chromosome-arm CNAs, whole-chromosomal CNAs, and WGDs always act as drivers, the location of focal CNAs and SNVs determines their selective potential. The relative rates for driver and passenger focal CNAs are denoted by p^d_{focal} and p^p_{focal}, respectively, and are defined analogously for SNVs.

S.5 Viability checkpoints

To ensure stability in the fitness landscape, each newly mutated cell c is subject to a number of viability checkpoints. Failure to adhere to any checkpoint results in the immediate death of c. By default, the viability checkpoints are as follows:

- $p_c < p_{\min}$.
- $p_c > p_{\max}$.
- Copy number in any region exceeds $CN_{\max}$.
- Number of SNVs in any region exceeds $SNV_{\max}$.

- Max number of mutated drivers exceeds $NMutDriver_{\max}$.

Here, $p_{\min}$, $p_{\max}$, $CN_{\max}$, $SNV_{\max}$, and $NMutDriver_{\max}$ are all predetermined parameters. The last parameter, $NMutDriver_{\max}$, is defined differently for the chromosome-arm selection model compared to the region and hybrid model. In the former case, each chromosome-arm is itself a 'driver' and is considered mutated if the number of regions is $< 0.5L$ or $> 1.5L$, where L is the number of regions in the unmutated arm. In the latter case, all OG, TSG, and EG regions are drivers and are considered mutated if the copy number is not equal to 2^{1+nWGD}, where $nWGD$ is the number of whole-genome duplications undergone in the cell's lineage.

S.6 Cell migration

After the birth-death process, cells undergo potential migrations to new or existing anatomical sites. Let A denote the total number of potential sites, and let c be a cell in site a at generation t. Migration probabilities are defined by a migration matrix $\alpha_c : A \times A$, where $\alpha_c(a, b)$ is the probability that c migrates from site a to site b, and encodes the pairwise distances between anatomical sites. In particular, the migration probabilities are defined as

$$\alpha_c(a,b) = \left\{ \begin{array}{ll} 0 & \text{if } a = b, \\ \frac{1}{d(a,b)} \cdot \epsilon & \text{otherwise.} \end{array} \right\}$$

where $d(a, b)$ is the distance between sites a and b and ϵ is a fixed per-generation baseline probability. Here, the migration probability scales inversely with distance. We explore two distance models which can be used individually or combined.

In the first model, named the 'static' model, $d(a, b)$ is static for each cell at every generation and can be provided by the user or generated randomly. This can be interpreted as an organotropism term, for example representing the physical distance in the body, similarities in microenvironments, or diffusion pathways. To generate random distance, we do as follows: or every site a, select a random point in a grid bounded by $(0, 0), (1, 1)$ and compute the euclidean distance d between each pair of points. The distances are normalized to be between 0 and 1, where 1 is the distance between the two farthest points.

In the second model, named the 'genotype' model, the distance is defined according to the cell's genotype. In particular, distance is defined as

$$d(a,b) = \left(\log \frac{s_b(c)}{\hat{s}_b} \right)^{-1}.$$

In other words, the probability that cell c successfully migrates to a site b increases as the cell acquires beneficial mutations. When used in conjunction with site-specific selection libraries, migration probabilities can also reflect compatibility with the the fitness landscape b.

S.7 Simulation algorithm

As the number of cells increases to what is expected upon diagnosis and sampling (around 10^6-10^9 cells), the runtime of simulating the tumors becomes impractical. To improve efficiency, we approximate the birth-death and migration process by simulating at the clonal level instead of at the single-cell level. Here, a clone is defined as the set of cells which have an identical set of driver mutations. As the birth, death, and migration probabilities depend only on the set of driver mutations, we can efficiently compute the cell counts of each clone at every generation. However, because agents are defined at the clonal level, we cannot track the accumulation of passenger mutations while simulating tumor growth and metastasis top-down. Instead, we introduce a second stage to the simulation that reintroduces passenger mutations bottom-up following the coalescent model. In the following section, we describe the two-stage simulation algorithm in more detail.

Clonal Evolution. Here we show how the clonal populations change from generation t to generation $t + 1$ under birth+death and migrations.

For clone w in site a after generation t, we denote its population size by $N_w(t)$ and replication rate by $p_w(t)$. At the beginning of generation $t+1$, π_b cells in w divide while the remaining $\pi_d = N_w(t) - \pi_b$ cells die:

$$\pi_b, \pi_d \sim \text{Multinomial}(N_w(t); p_w(t), p_w(t) - 1).$$

Of the π_b cells that divide, $\hat{\pi}_b$ acquire at least one driver mutation, becoming new clones, while the remaining $\breve{\pi}_b = \pi_b - \hat{\pi}_b$ do not and thus stay in w. For convenience, we define z to be the probability of a driver mutation occurring:

$$z = 1 - \big((1 - p^{driv})(1 - p^{chrom})(1 - p^{arm})(1 - p^{wgd})\big)$$

$$\hat{\pi}_b, \breve{\pi}_b \sim \text{Multinomial}(\pi_b; z, 1 - z).$$

At this point, we update the number of cells remaining in w to be $N_w(t) \leftarrow 2\breve{\pi}_b - \hat{\pi}_b$, and initialize $\hat{\pi}_b$ new clones each with 1 cell.

In the second stage of the generation, following the birth-death process, are potential migrations. For clone w in site a, let $\hat{\pi}_m$ be the number of cells which migrate away from a and $\breve{\pi}_m = N_w(t) - \hat{\pi}_m$ be the number of cells which migrate away from a. For any site b, let $\alpha_{a,b}(w)$ be the probability that cells in w migrate to site b. If there are A total sites labeled $1, ..., A$, then we have

$$\hat{\pi}_m^1, ..., \hat{\pi}_m^A \sim \text{ Multiinomial}(N_w(t); \alpha_{a,1}(w), ..., \alpha_{a,A}(w)),$$

with $\hat{\pi}_m = \sum_{b=1,...,A} \hat{\pi}_m^b$.

To finalize the clone population counts for generation $t+1$, we set $N_w(t+1) \leftarrow N_w(t) - \hat{\pi}_m$ and for any b with $\hat{\pi}_m^b > 0$, we initialize a new clone w' in b with $\hat{\pi}_m^b$ cells.

Initialization and Termination. We begin the birth-death-migration process at generation $t = 1$ with a single clone w in the primary site and $N_w(1) = 1$. The process terminates once all A anatomical sites have at least $\mathcal{N}$ cells or the current generation surpasses t_{max}.

S.8 Cell Sampling and Phylogeny Construction

After termination of the clonal evolution process at generation $\hat{t}$, for each site a we sample a given number of cells randomly from the clones present in a at $\hat{t}$ with probability proportional to clone cell count. Once the set of observed cells is determined, we next construct a clone tree $\mathcal{T}$, migration graph G, and (possibly) a single-cell lineage tree T. We begin by constructing the clone tree $\mathcal{T}$ as follows. First, T is initialized to contain the set of clones from any site with at least one observed cell as vertices. Second, for each observed clone, each ancestral clone is added as a vertex (if not already present) with a corresponding edge by traversing backwards in time until the initial clone used to seed the primary site is reached.

Following [5], the migration graph G is derived directly from $\mathcal{T}$ by collapsing all vertices with the same anatomical site labels into a single vertex and removing any self-edges. To construct the cell lineage tree T, we fully binarize $\mathcal{T}$ and assign branch lengths such that the ancestral cells at bifurcations and multifurcations are sampled uniformly from the larger clonal population. Note that branch lengths correspond to the number of generations, which is used to determine the number of passenger mutations along each edge. We update the topology and passenger counts as follows. Let the children of clone w present in $\mathcal{T}$ be denoted by $ch(w)$ (descendants separated by one branch) and its parent by $par(w)$. The birth generation of clone w is denoted b_w. In a top-down traversal from the founder clone, if $|ch(w)| > 1$, execute procedure *resolve_multifurcation* provided below. In summary, consider some node w in the clone tree with more than one child and denote its children $ch(w) = \{w'_1, w'_2, ..., w'_n\}$, ordered by birth generation so that w'_n was born latest. We iterate backwards in time from the birth generation of the most recent child, $b_{w'_n}$, down to the birth generation of w, b_w, and at each iteration two operations occur on a mutable set of descendants, initially empty. First,

if the birth generation of some child from $ch(w)$ equals the generation of the current iteration, it is added to the descendants set. Second, for each node in the descendants set, it is randomly assigned to a 'bucket', with the number of buckets equal to the recorded cell count of the previous generation. If two nodes are assigned to the same bucket, it corresponds to these nodes sharing an intermediate common ancestor. In this instance, the nodes are removed from the descendants set, pruned from node w, and added as children to a new intermediate node which is regrafted as a child of w and inserted into the descendants set.

To add passenger mutations, we perform a top-down traversal of T (or $\mathcal{T}$ if only generating a clone tree) and draw a number of segmental CNAs and SNVs from per-generation passenger mutation rates. Note for segmental CNAs, the location and length are restricted so as to not interfere with downstream driver mutations.

```
procedure RESOLVE_MULTIFURCATION(parent, children, popsizes)
    groups ← []
    id ← 0
    t_0 ← b_parent
    t_max ← max {b_child | child ∈ children}
    for t = t_max ... t_0 do
        draws ← dict(array)
        for g ∈ groups do
            x ← Unif(0, popsizes[t])
            while |draws[x]| > 2 do
                Redraw x
            Append g to draws[x]
        for group ∈ draws.values do
            if |ngroup| > 1 then
                Create new node n and set b_n = t
                Set ch(n) ← group
                Set par(g) = n for each g ∈ groups
                Remove each g ∈ group from groups
                Add n to groups
        for child ∈ children do
            if b_child = t then
                Add child to groups
                Remove child from children
    Set ch(parent) = []
    for g ∈ groups do
        Append g to ch(parent)
        Set par(g) = parent
```

S.9 Data Generation

SISTEM generates three types of observed output data based on the simulated clone tree or single-cell lineage tree: mutation profiles, read counts, and DNA-seq reads. Mutation profiles are separate for SNVs and CNAs, and can be generated for observed cells and clones, as well as ancestral clones. To generate copy number profiles (CNPs), for each cell/clone we first compute region-level copy numbers by iterating across all strands of each allele-specific chromosome and totaling the number of occurrences of each reference region. Then, the region-level copy numbers are (optionally) grouped into larger bins, with bin-level copy numbers equaling the average of all region-level copy numbers. SNV profiles consist of bi-allelic genotypes for each mutated locus (SNV) occurring on any observed cell/clone. To generate the genotype of some locus j occurring in region i of chromosome k, for each cell/clone, the genotype of allele A (resp. B) is equal to the non-reference base-pair if present, otherwise it is equal to the reference base pair. If no copies of region i exist, then the genotype is listed as '-'. Note that the reference base pairs can be chosen randomly or can

be determined by a provided reference genome.

Read counts are generated differently for clones and single cells. Clonal read counts consist of the number of reads covering the reference and alternate alleles of each SNV generated from separate bulk sequencing samples for each anatomical site. For site a, let the observed clones present in a be denoted $w_1, w_2, ..., w_V$. For each observed clone w_v, we define its proportion to be $\tau(w_v) = S(w_v)/\sum_{r'} S(w_{v'})$, where $S(w_v)$ is the number of cells sampled from w_v during the sampling phase. Note that the samples can be diluted with normal cells in the previous step. For some SNV j occurring on region i of chromosome k, let $\hat{x}^v_{k,i}$ and $\check{x}^v_{k,i}$ be the number of copies of region i in w_v which contain the reference and alternate base pair, respectively. Note the total copy number $x^v_{k,i} = \hat{x}^v_{k,i} + \check{x}^v_{k,i}$. We compute the frequency $f_{a,j}$ of mutation j in site a as follows:

$$f_{a,j} = \sum_{r=1}^{V} \tau(w_v) \cdot \frac{\check{x}^v_{k,i}}{x^v_{k,i}}.$$

In other words, $f_{a,j}$ is the proportion of reads sampled from site a which contain SNV j. Note that a small constant ($1e-8$) is added to the denominator to prevent division by 0 if both copies are lost. To generate total and variant read counts, we first compute the expected total read count $r'_{a,j} = C \cdot \sum_v^V x^v_{k,i}/2V$, where C is a baseline coverage parameter. The total read count $r_{a,j}$ and variant read count $q_{a,j}$ are then generated as follows:

$$\begin{aligned} r_{a,j} &\sim \text{Poisson}(r'_{a,j}) \\ q_{a,j} &\sim \text{Binomial}(r_{a,j}, f_{a,j}), \end{aligned}$$

For single cells, for each cell the mutated genome is first expanded into two complete sequences (one for each allele) by replacing region ids with their corresponding DNA segment from the reference genome. Select base pairs in the reference DNA segments are updated to reflect the presence of SNVs in the mutated genome. The user may pass a separate paternal and maternal reference genome used to substitute the regions on the paternal (resp. maternal) allele. Otherwise, a single haploid reference can be provided for both alleles. Next, for each allele-specific chromosome, read counts are generated for every region on each strand, drawn from a Poisson distribution with mean $r = \frac{M \cdot C}{2 \cdot L}$, where M is the region length, C is the coverage, and L is the read length. Read counts belonging to copies of the same reference region are then summed together to create a single read count for each allele-specific reference region.

SISTEM is also able to generate raw paired-end single-cell DNA-sequencing reads by integrating the short read simulator DWGSIM (`https://github.com/nh13/DWGSIM`). For each region on every mutated chromosome strand, the readcount and mutated sequence created in the previous step are passed to DWGSIM, which also allows for a tunable sequencing error parameter.

S.10 Evaluation of tumor cell lineage tree inference methods using simulated single-cell CNA profiles

A common approach to studying the genetic heterogeneity within a tumor is to construct a phylogenetic tree based on single-cell DNA-sequencing (scDNA-seq) data for cells sampled from the tumor. This results in a cell lineage tree for the tumor, in which leaves correspond to sampled/observed cells and internal nodes correspond to hypothetical ancestral cell populations. Tumor cell lineage trees are useful for identifying lineage expansions and associated driver mutations, estimating the timing of mutations, and identifying subclonal populations [9]. However, the inference of such tumor cell lineage trees is a challenging problem whose difficulty is exacerbated by the low resolution and high error typical of scDNA-seq. Since the ground truth cell lineage tree is unavailable for real datasets, simulations are commonly used to assess and benchmark the performance of tumor cell lineage tree inference methods. SISTEM can significantly improve such assessment and benchmarking by (i) allowing for the creation of more realistic simulated datasets that better capture the underlying biological processes driving tumor growth and evolution, and (ii) enabling more fine-grained evaluation of methods along new dimensions related to selection, fitness, migration, etc., using the

many new evolutionary models and parameters available in SISTEM but not available in existing simulation frameworks. In the example application, we explore the impact of one such evolutionary parameter, *passenger CNA rate*, on the accuracy of cell lineage tree reconstruction. In particular, we assess the performance of DICE-bar [10], a recently published state-of-the-art distance-based method for reconstructing tumor cell lineage trees using CNA profiles at single-cell resolution.

The passenger CNA rate controls the rate at which non-driver focal CNAs occur during cell replication. Thus, a higher passenger CNA rate leads to greater numbers of non-driver focal (segmental) gains and deletions throughout the cell lineage tree. Importantly, this parameter does not affect the rate of driver focal CNAs, which determines the overall shape of the cell lineage tree and the evolutionary trajectories of individual cell lineages. It is therefore interesting to see how the ability to reconstruct accurate cell lineage trees is affected as the rate of non-driver focal CNAs increases, while keeping the rate of driver focal CNAs constant (at its default value). For this evaluation, we generated 100 single-tumor simulation instances using the chromosome-arm selection model with selection coefficients drawn from Uniform$(-0.25, 0.25)\}$. Termination occurred when the tumor population reached 5×10^6 cells. For each simulation, a separate dataset was generated for each of the 50 different passenger CNA rates $\in \{0.01, 0.02, \ldots, 0.5\}$, and 100 cells were randomly sampled. This resulted in a total of 5000 simulated datasets, each with 100 cells.

We applied DICE-bar to the simulated CNPs of the cells in each dataset. We first compared the accuracies of the inferred cell lineage trees to the corresponding ground truth cell lineage trees using the normalized Robinson-Foulds distance (NRFD) [8], assuming both trees to be unrooted. This metric computes the number of bipartitions that differ between the two trees, normalized so that a value of 0 indicates the trees are identical while a value of 1 indicates they are maximally different. As expected, the NRFD of the DICE-bar trees decreases rapidly as the passenger CNA rate increases (Supplementary Figure S1a). This is because a higher rate of passenger CNAs leads to more variation between different cells within the tumor, making phylogenetic inference easier. However, we find that increasing the passenger CNA rate offers diminishing improvements to the accuracy of the DICE-bar trees; for example, the difference between the mean NRFD for passenger CNA rates 0.1 and 0.2 is 0.156, compared to just 0.061 between passenger CNA rates 0.3 and 0.4. Thus, further increases in the passenger CNA rate are unlikely to substantially improve cell lineage tree inference accuracy (Supplementary Figure S1a). This is likely due to the fact that, despite high rates of passenger CNAs, cells belonging to the same clone and separated by only a few generations are likely to have nearly identical CNPs, making it difficult to determine their placement relative to each other within the tree. In addition, given the limited resolution of the CNPs, an over-abundance of focal CNAs across the tree can lead to confounding instances of parallel and back mutations.

We next evaluated the ability of the DICE-bar trees to recover subclonal populations present in the sampled cells. Following [10], we used the Clone F_1 score to measure clone inference accuracy. Specifically, for a given ground truth clone, represented by a subset of sampled cells, we identify the clade in the reconstructed cell lineage tree with maximum F_1-score with respect to that ground truth clone. We then average these F_1-scores across all clones present in the ground truth cell lineage tree. As Supplementary Figure S1b shows, the mean Clone F_1 score is largely unaffected by the passenger CNA rate and remains consistently high across all 50 passenger CNA rate values. These results show that clonal populations can be reliably identified even when passenger CNA rates are very low, as long as the clones differ sufficiently in their driver mutations.

This example application demonstrates the utility of SISTEM for systematically evaluating tumor phylogeny inference methods under controlled evolutionary scenarios. Our analysis shows that DICE-bar's performance benefits from higher rates of passenger CNAs, but with diminishing returns at higher rates. The analysis also shows that DICE-bar's ability to recover subclonal structure remains consistently strong irrespective of passenger CNA rate.

S.11 Creating simulated datasets following cancer subtype-specific mutation patterns

Outside of their primary anatomical location, cancer subtypes can be uniquely characterized by tumor-specific aneuploidy patterns [6]. These patterns may also be used to distinguish within the same subtype,

possibly between primary and metastatic tumors. The forces that promote gains and losses of individual chromosome arms are in part governed by selective pressures of the diverse tissue microenvironments within the body; however, a general theory explaining how aneuploidy patterns emerge remains incomplete [3]. Simulation models parameterized to mimic empirically-derived mutation patterns offer one approach to study the growth and evolutionary behaviors of specific cancer subtypes, as well as to evaluate inference methods under tailored experimental settings. In this section, we demonstrate how SISTEM can be used to generate simulated datasets parameterized by cancer subtype-specific mutation patterns derived from real cancer data. For this demonstration, we show how to scale selection coefficients under the chromosome-arm selection model according to the frequencies of chromosome arm-level CNAs observed in a set of real samples sharing the same cancer subtype.

Mutation patterns (described by CNA frequencies) were obtained from the MSK-MET pan-cancer cohort [7], a large multi-omic dataset that includes genomic data from more than 25,000 samples across 50 cancer types, including samples from both primary and metastatic tissues. To characterize the aneuploidy patterns of each sample, log-scaled genome-wide copy number ratios were derived as described in [2], which we averaged across each chromosome arm to overcome both sparsity and non-uniform segment boundaries. The samples were then grouped according to their cancer subtype and the anatomical location of the sample (for example, pancreatic adenocarcinoma sampled from the liver constitutes a group). We next determined the mutation pattern of each group by computing the frequency of chromosome arm-level gains (resp. losses), computed for each arm as the number of samples in a group which undergo an arm-level gain (resp. loss) divided by the total number of samples in the group. We say sample i undergoes an arm-level gain (resp. loss) if the average copy number along that arm is $\geq p_i + 0.5$ (resp. $\leq p_i - 0.5$), where p_i is the cell ploidy. To better highlight the significant frequencies and ignore noise, we set both gain and loss frequencies of an arm to 0 if they are within 0.1 of one another.

We next describe how SISTEM can be used to generate simulated datasets fitted to a particular mutation pattern. Let f_a^g and f_a^l be the respective gain and loss frequencies of chromosome-arm a according to the mutation pattern. Under the chromosome-arm selection model, we set the selection coefficient δ_a of each chromosome-arm a as follows:

$$\delta_a = \left\{ \begin{array}{cc} s * f_a^g & \text{if } f_a^g > f_a^l, \\ -s * f_a^l & \text{otherwise.} \end{array} \right\},$$

where s is a linear scale parameter. Note that the selection coefficient is positive (resp. negative) if the gain rate is greater than (resp. less than) the loss rate. Here we set $s = 0.25/f^{max}$ so that the arm with the maximum CNA frequency has a selection coefficient of 0.25. With this approach, large-scale CNAs which result in aneuploidy patterns similar to those which are empirically-derived will be selected for.

For brevity, we highlight the results from two large groups: primary Colorectal MSS samples and primary Ovarian High-Grade Serous Carcinoma samples, totaling 2090 and 350 samples, respectively. These groups were chosen because they both contained a large number of samples, and had a relatively high number of chromosome arms with significant event frequencies compared to other groups. The number of significant frequencies in each group (chromosome arms a such that $|f_a^g - f_a^l| >= 0.1$) numbered 15/44 and 27/44, respectively, with the largest frequencies in each group being $f_{chr17p}^l = 0.61$ and $f_{chr20q}^g = 0.48$ (allosomes are excluded). For each group, we generated a corresponding "simulated" group consisting of 100 simulation instances with chromosome-arm selection coefficients computed using the process described above. For comparison, we also generated a "Random" group which utilized random selection coefficients. Termination occurred when the tumor population reached 5×10^6 cells, after which chromosome arm-level CNA frequencies were computed for each simulated group. All other parameters were set to their defaults.

A comparison between the empirically-derived (true) and simulated mutation patterns for the Colorectal samples is visualized in Supplementary Figure S2. As expected, we find a clear correlation between the true and simulated mutation patterns for both groups. To quantify this correlation, we computed the Pearson Correlation Coefficient (PCC) between the true and simulated chromosome arm-level CNA frequencies. To ensure an appropriate comparison, we multiply all loss rates by -1 so that all values appear in the range (-1, 1). The PCC between the true and simulated Colorectal and Ovarian groups support a high correlation

with values of 0.908 and 0.849, respectively. By contrast, the PCC between the true Colorectal and Ovarian groups and the Random group show values of 0.066 and -0.094, respectively. We next compared the values of the frequencies themselves by computing the Mean-Square Error (MSE). We find the MSE between the true and simulated Colorectal and Ovarian groups are relatively low with values of 0.034 and 0.049, respectively. The MSE value between the true Colorectal and Ovarian groups and the Random group is much higher at 0.11 in both instances.

These results demonstrate how SISTEM can mimic the mutation patterns for specific sets of samples using easily-computed summary statistics and a simple linear scale. It should be noted that under the SISTEM model, the chromosome arm-level CNA frequencies used as summary statistics in this experiment are influenced by a multitude of parameters beyond selection coefficients (for example, mutation rates, number of generations, etc.). While default parameter values will result in reasonably close mutation patterns in the simulated data, a dedicated simulation-based parameter inference model would be considerably more robust and accurate, and is a promising direction for future work.

Supplementary Figures

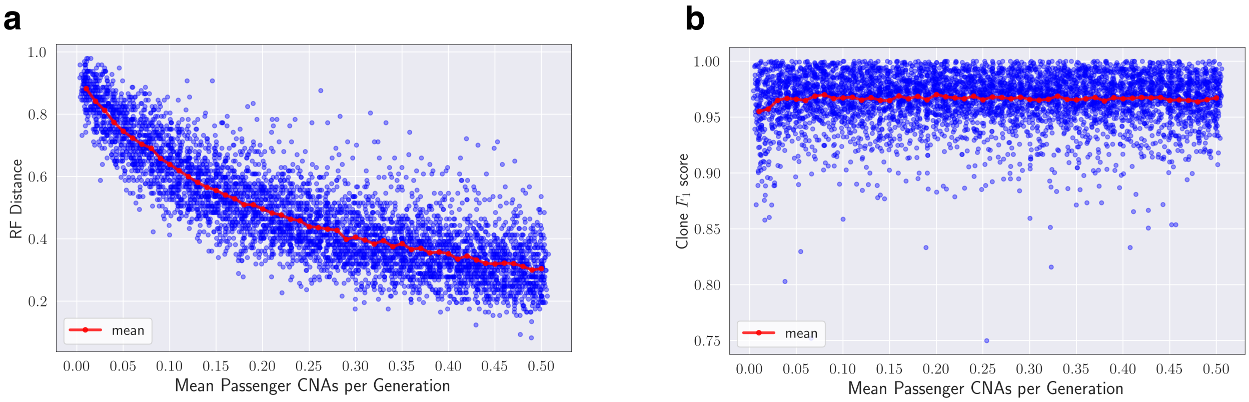

Figure S1: **Accuracy of tumor cell lineage trees inferred using DICE-bar.** The plots show the effect of passenger CNA rate on the accuracies of reconstructed DICE-bar cell lineage trees. Results are shown for 50 different CNA rates in the range $[0.01, 0.5]$, with 100 datasets for each rate. **(a)** Normalized Robinson-Foulds Distance (NRFD) between DICE-bar trees and corresponding ground truth cell lineage trees. Smaller values of NRFD imply greater reconstruction accuracy. **(b)** Clone inference accuracy of the DICE-bar trees measured in terms of Clone F_1 score. Larger values of Clone F_1 score imply greater clone inference accuracy.

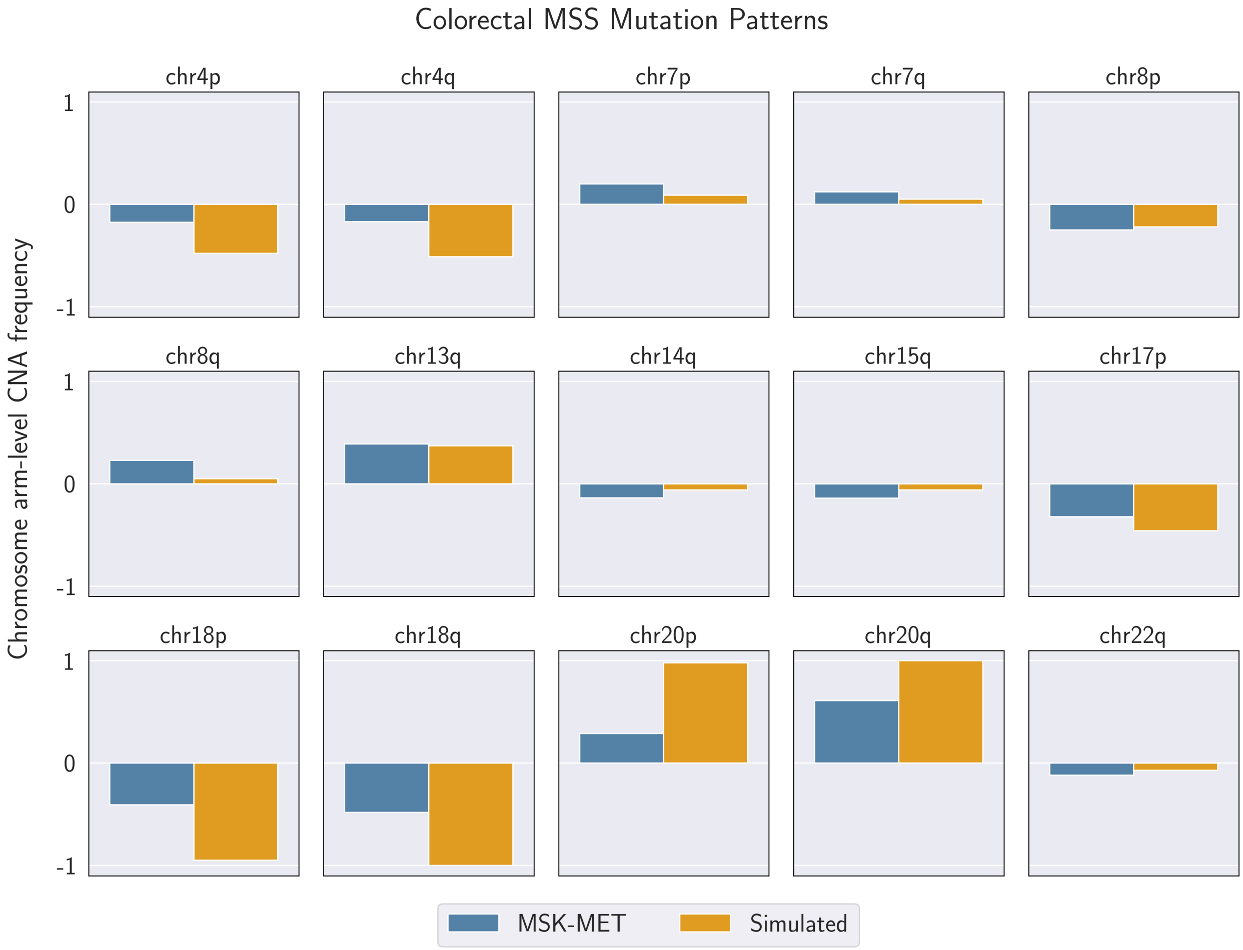

Figure S2: **Empirical vs simulated aneuploidy patterns of Colorectal MSS.** A visualization of the chromosome arm-level CNA frequencies (mutation patterns) derived empirically from 2090 primary Colorectal MSS samples in the MSK-MET cohort dataset (blue) compared to 100 simulation instances with selection coefficients parameterized according to the frequencies (orange). A positive value corresponds to the arm-level CNA gain rate, while a negative value corresponds to the loss rate (the larger of the two is shown). Only those chromosome arms with "significant" CNA rates (the absolute difference between the gain rate and loss rate is ≥ 0.1) are shown.

References

[1] L. Bányai, M. Trexler, K. Kerekes, O. Csuka, and L. Patthy. Use of signals of positive and negative selection to distinguish cancer genes and passenger genes. *Elife*, 10:e59629, 2021.

[2] D. T. Cheng, T. N. Mitchell, A. Zehir, R. H. Shah, R. Benayed, A. Syed, R. Chandramohan, Z. Y. Liu, H. H. Won, S. N. Scott, et al. Memorial sloan kettering-integrated mutation profiling of actionable cancer targets (msk-impact): a hybridization capture-based next-generation sequencing clinical assay for solid tumor molecular oncology. *The Journal of molecular diagnostics*, 17(3):251–264, 2015.

[3] T. Davoli, A. W. Xu, K. E. Mengwasser, L. M. Sack, J. C. Yoon, P. J. Park, and S. J. Elledge. Cumulative haploinsufficiency and triplosensitivity drive aneuploidy patterns and shape the cancer genome. *Cell*, 155(4):948–962, 2013.

[4] K. N. Dinh, I. Vázquez-García, A. Chan, R. Malhotra, A. Weiner, A. W. McPherson, and S. Tavaré. CINner: modeling and simulation of chromosomal instability in cancer at single-cell resolution. *PLOS Computational Biology*, 21(4):e1012902, 2025.

[5] M. El-Kebir, G. Satas, and B. J. Raphael. Inferring parsimonious migration histories for metastatic cancers. *Nature genetics*, 50(5):718–726, 2018.

[6] A. Laughney, S. Elizalde, G. Genovese, and S. Bakhoum. Dynamics of tumor heterogeneity derived from clonal karyotypic evolution. *Cell Reports*, 12(5):809–820, 2015.

[7] B. Nguyen, C. Fong, A. Luthra, S. A. Smith, R. G. DiNatale, S. Nandakumar, H. Walch, W. K. Chatila, R. Madupuri, R. Kundra, et al. Genomic characterization of metastatic patterns from prospective clinical sequencing of 25,000 patients. *Cell*, 185(3):563–575, 2022.

[8] D. Robinson and L. Foulds. Comparison of phylogenetic trees. *Mathematical Biosciences*, 53(1):131–147, 1981.

[9] R. Schwartz and A. A. Schäffer. The evolution of tumour phylogenetics: principles and practice. *Nature Reviews Genetics*, 18(4):213–229, Apr 2017.

[10] S. Weiner and M. S. Bansal. DICE: fast and accurate distance-based reconstruction of single-cell copy number phylogenies. *Life Science Alliance*, 8(3), 2025.

[11] Y. H. Woo and W.-H. Li. Dna replication timing and selection shape the landscape of nucleotide variation in cancer genomes. *Nature communications*, 3(1):1004, 2012.
